# The Interplay between Two Transcriptional Repressors and Chaperones Orchestrates *Helicobacter pylori* Heat-Shock Response

**DOI:** 10.3390/ijms19061702

**Published:** 2018-06-07

**Authors:** Davide Roncarati, Vincenzo Scarlato

**Affiliations:** Department of Pharmacy and Biotechnology (FaBiT), University of Bologna, 40126 Bologna, Italy; vincenzo.scarlato@unibo.it

**Keywords:** *Helicobacter pylori*, heat-shock response, transcriptional regulation, HspR repressor, HrcA repressor

## Abstract

The ability to gauge the surroundings and modulate gene expression accordingly is a crucial feature for the survival bacterial pathogens. In this respect, the heat-shock response, a universally conserved mechanism of protection, allows bacterial cells to adapt rapidly to hostile conditions and to survive during environmental stresses. The important and widespread human pathogen *Helicobacter pylori* enrolls a collection of highly conserved heat-shock proteins to preserve cellular proteins and to maintain their homeostasis, allowing the pathogen to adapt and survive in the hostile niche of the human stomach. Moreover, various evidences suggest that some chaperones of *H. pylori* may play also non-canonical roles as, for example, in the interaction with the extracellular environment. In *H. pylori*, two dedicated transcriptional repressors, named HspR and HrcA, homologues to well-characterized regulators found in many other bacterial species, orchestrate the regulation of heat-shock proteins expression. Following twenty years of intense research, characterized by molecular, as well as genome-wide, approaches, it is nowadays possible to appreciate the complex picture representing the heat-shock regulation in *H. pylori*. Specifically, the HspR and HrcA repressors combine to control the transcription of target genes in a way that the HrcA regulon results embedded within the HspR regulon. Moreover, an additional level of control of heat-shock genes’ expression is exerted by a posttranscriptional feedback regulatory circuit in which chaperones interact and modulate HspR and HrcA DNA-binding activity. This review recapitulates our understanding of the roles and regulation of the most important heat-shock proteins of *H. pylori*, which represent a crucial virulence factor for bacterial infection and persistence in the human host.

## 1. Introduction

When bacterial cells experience a sudden temperature increase, they promptly induce the expression of a group of genes known as heat-shock genes. They encode a class of proteins, referred to as heat-shock proteins (HSP), which are highly conserved throughout all kingdoms of life [1]. This mechanism of cellular protection, crucial for survival and adaptation to adverse environmental growth conditions, is known as heat-shock response. In addition to temperature upshift, heat-shock proteins typically accumulate following different kinds of stress insults and are involved in several cellular processes, including promoting the correct folding of newly-synthesized polypeptides, preventing aggregation of cellular proteins under stress conditions, and recovering proteins that have been partially or completely unfolded by stresses [2]. During adverse conditions of growth, in fact, spontaneous folding of nascent polypeptides is inefficient and error-prone, and a predominant fraction of newly-synthetized proteins undergoes a chaperone-assisted folding process. Furthermore, a significant fraction of already-folded proteins gets partially or completely denatured and becomes incline to form amorphous aggregates [3]. The heat-shock response is universally conserved in both prokaryotic and eukaryotic cells and the major HSP and chaperones are among the most conserved proteins known so far. Nonetheless, the molecular mechanisms underpinning the regulation of heat-shock genes’ expression in response to changing environmental conditions are extremely diversified even among different bacterial species. In particular, bacteria usually exploit combinations of transcriptional, as well as posttranscriptional, regulatory mechanisms to control the rapid accumulation of HSP only when they are necessary.

*Helicobacter pylori* is one of the most widespread and successful human pathogens, infecting the gastric mucosa of about half of the population in the world. In order to establish a persistent infection, *H. pylori* exploits several virulence factors, defined as the effectors that permit the bacterium to contact, invade and persist into the host and respond to the extremely adverse conditions typical of the human stomach [4]. Among *H. pylori* virulence factors, that include the flagellar apparatus [5], the urease enzyme [6], a collection of adhesion molecules [7], and the toxic effectors VacA and CagA [8,9], the highly conserved class of stress-induced HSP has to be included [10]. These proteins, through their canonical roles in protecting cellular proteins and in maintaining cellular homeostasis, allow the pathogen to adapt and survive in the hostile niche represented by the human stomach. Heat-shock proteins are also expressed by gastric epithelial cells of the human host, where they act as intracellular molecular chaperones, helping the eukaryotic cells to maintain protein homeostasis. Although bacterial infection is generally associated with increased host HSP expression [11], experimental evidence suggests that *H. pylori* infection is associated with decreased expression of major HSP, such as HSP60 and HSP70 [12,13,14,15].

The present review is focused on the heat-shock response of *H. pylori*, and especially on the strategies adopted by this important human pathogen to finely modulate the expression and accumulation of the most important HSP upon the perception of stress signals. Considering the central role played by HSP in *H. pylori* virulence and pathogenesis, the deep comprehension of the mechanisms at the basis of heat-shock regulation is of crucial importance and could provide new ideas for the development of novel antibacterial strategies.

## 2. Transcriptional Regulation of Heat-Shock Genes in *H. pylori*

**Figure 1:**
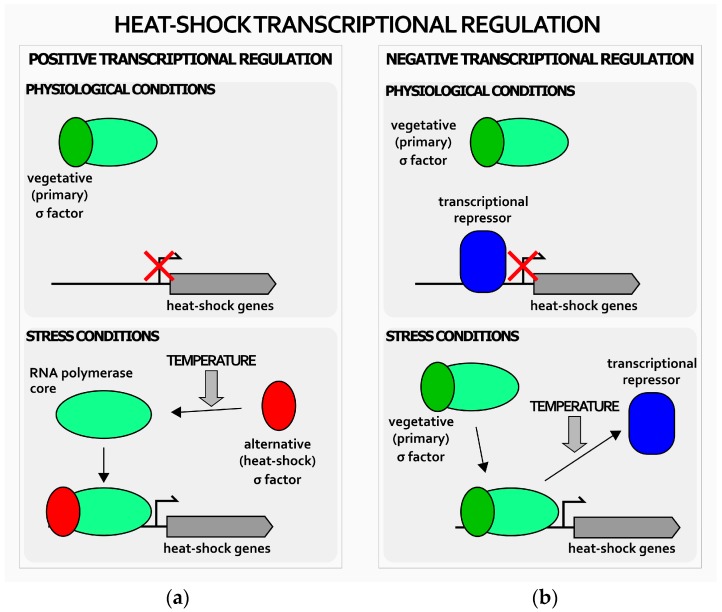
Schematic representation of positive and negative mechanisms of heat-shock genes transcriptional regulation in bacteria. Positive transcriptional regulation (**a**) relies on heat-shock alternative sigma factor (red oval) that, upon interaction with the core RNA polymerase (green oval), directs the transcription enzyme on heat-shock genes’ promoters, thereby activating their transcription; negative regulation (**b**) is based on repressors (blue oval) that keep transcription of heat-shock genes repressed during normal growth conditions. Upon heat-shock, they detach from heat-shock genes’ promoters, whose transcription is operated by the RNA polymerase associated to the primary σ factor (green oval). Bent arrow indicates the transcription start site.

In general, heat-shock genes’ transcriptional regulation in bacteria can be either positive or negative (Figure 1). Positive transcriptional regulation is based on condition-specific alternative sigma (σ) factors (the subunit of the RNA polymerase that provides promoter specificity to the multi-subunit enzyme) able to outcompete the vegetative σ factor normally associated with the RNA polymerase. The heat-shock σ factor, once engaged by a temperature increase from 37 to 42 °C, associates to the RNA polymerase and directs the transcription machinery to specific, heat-shock related promoters, thereby reprogramming cellular transcription (Figure 1a) [1,2].

This positive mechanism of transcriptional regulation is widely distributed among bacteria, including pathogenic and opportunistic bacteria, like *Vibrio cholerae* and *Pseudomonas aeruginosa* [16,17], although the model organism, *Escherichia coli*, where the heat-shock sigma factor, σ^32^, governs heat-shock genes’ activation, remains the best-characterized example [18]. 

**Figure 2:**
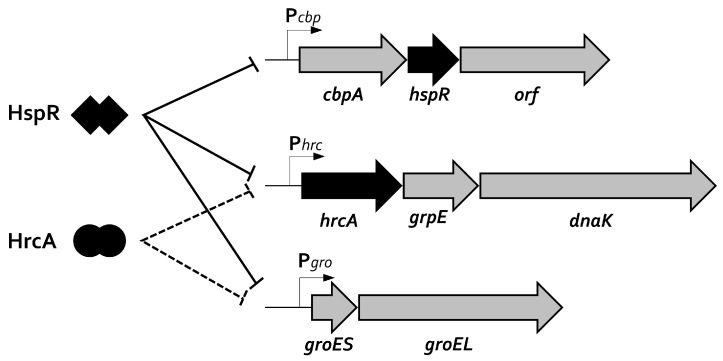
Schematic representation of the multi-cistronic operons containing the major heat-shock proteins of *H. pylori*. The HspR regulator represses alone the transcription of its own *cbpA-hspR-orf* operon, while both HspR and HrcA repressors combine to control the expression of *groEL-groES* and *hrcA-grpE-dnaK* operons. HspR and HrcA DNA-binding and transcriptional repression are represented by solid and dotted lines, respectively.

In sharp contrast, negative transcriptional regulation is managed by dedicated repressors, whose DNA-binding activity changes in response to fluctuating environmental temperature. Specifically, under normal conditions of growth, these repressors bind specific operators and repress heat-shock genes’ transcription. Upon temperature upshift (Figure 1b), repressors detach from their operators and transcription is rapidly induced by the RNA polymerase associated to the housekeeping σ subunit [1,2]. In several bacterial species, heat-shock transcriptional regulation is managed exclusively by dedicated transcriptional repressors, including clinically-relevant pathogens, like *Streptococcus pneumoniae*, *Micoplasma genitalium*, and *Clostridium difficile* [19,20,21]. The analysis of the *H. pylori* genome highlighted the lack of a homologue of the *E. coli* heat-shock sigma factor, σ^32^. In contrast, this gastric pathogen expresses two genes encoding two distinct transcriptional repressors, which are homologues to *Bacillus subtilis* HrcA and to *Streptomyces coelicolor* HspR [22,23,24]. HrcA and HspR encoding genes belong to multi-cistronic operons containing other important heat-shock genes, as detailed in Figure 2. 

Initial studies aimed at the characterization of HspR and HrcA contribution to heat-shock genes’ regulation date approximately 20 years ago. In particular, it was demonstrated that *hspR* gene disruption led to the constitutive high-level transcription of the two *cbpA-hspR-helicase* and *hrcA-grpE-dnaK* heat-shock operons. In addition, *hspR* inactivation provokes the accumulation of the bi-cistronic *groES-groEL* operon, coding for the multi-subunit chaperonin GroE [25]. Using a similar approach, it was demonstrated that *hrcA* inactivation affects the transcription of *groES-groEL* and *hrcA-grpE-dnaK* operons, while the expression of the HspR encoding operon appears to be unaffected [26]. Following these observations, a model in which HspR alone is able to repress its own transcription was proposed, while both heat-shock regulators HspR and HrcA are necessary to repress transcription of the *groES-groEL* and *hrcA-grpE-dnaK* operons (Figure 2). This hypothesis was further confirmed by in vitro DNA-binding studies, demonstrating the direct interaction between the repressors and the promoters controlling heat-shock operons’ transcription. DNase I footprinting assays performed with purified recombinant HspR protein showed direct binding of the repressor to all three operons’ promoters, with important differences in the position of the binding sites, with respect to the core promoter elements, among the different heat-shock promoters [25,27]. In detail, whereas on the promoter of the *cbpA-hspR-helicase* operon, HspR occupies a region overlapping the −35 and −10 sequence elements (essential for the RNA polymerase promoter recognition and binding), the HspR binding sites on the promoters of *groES-groEL* and *hrcA-grpE-dnaK* operons map upstream of the core promoter regions, being centered 72 and 117 bp upstream from their specific transcription start sites, respectively [25,27]. It is interesting to note the extended binding site of HspR (around 75 bp), despite being a relatively small protein [27]. On the other hand, HrcA binds the *dnaK* and *groE* operons and the interaction involves DNA regions overlapping the core promoter elements and the transcription start sites, left unoccupied by its regulatory partner HspR [27,28]. For both HspR and HrcA repressors, the experimentally-identified operators on the heat-shock operons promoters comprise conserved sequences similar to the well-characterized HspR and HrcA recognition sequences, known as HAIR (for HspR Associated Inverted Repeat) and CIRCE (for Controlling Inverted Repeat of Chaperone Expression), respectively. These inverted repeats are located in the center of the protected regions mapped through DNase I-footprintings and are expected to represent the specific DNA recognition sites governing the repressors’ binding event, even though, so far, a formal demonstration of their role is still missing. Another point that needs further clarification is the role played by HspR and HrcA on co-regulated promoters. While, in fact, the position of HrcA DNA-binding sites is close to, or overlapping, the −35 and −10 promoter elements, HspR operators, on *groES-groEL* and *hrcA-grpE-dnaK* promoters, are located upstream of the core-promoter region. Moreover, even though HrcA and HspR binding sites are very close to each other and both repressors are necessary for full repression, they appear, at least in vitro, to bind independently their operators with no direct protein-protein interaction [27]. A possible explanation could be that in vivo binding of HrcA to its target sequences is not efficient without the assistance of a functional HspR repressor. 

The interplay between HrcA and HspR might be explained by hypothesizing the existence of chaperone-mediated protein-protein interactions between the two repressors, which may be a prerequisite for the formation of a stable repression-competent complex. The HrcA/HspR-mediated transcriptional repression operates at the physiological temperature of growth; upon a sudden temperature increase, repression is relieved and heat-shock genes are promptly transcribed with a very rapid kinetic. In detail, following the perception of a stress signal, the amount of the transcripts of the three heat-shock operons increases rapidly upon temperature upshift, starting at 2 min and reaching a maximum at 10 to 15 min treatment. Interestingly, the ratio of this increase of transcripts is different for P*gro*, P*hrc*, and P*cbp* promoters, ranging from five-fold for P*gro* to seven-fold for P*cbp*, and 11-fold for P*hrc*. The phase of maximal induction is followed by a decline or adaptation phase during which mRNA amounts gradually decrease to a new steady-state level. In addition to responding to a temperature upshift, the transcription of *H. pylori* heat-shock operons is induced by other stress signals, including high osmolarity and accumulation of misfolded proteins in the cytoplasm [29,30]. The expression pattern of heat-shock genes has been investigated also in response to other relevant stimuli encountered by *H. pylori* during infection and colonization of the human host. Specifically, Huesca and collaborators [31,32] demonstrated the induction of GroEL and DnaK proteins in *H. pylori* after acid shock, suggesting an important role of chaperones for survival under low-pH conditions. This aspect could be particularly relevant considering the change of intra-gastric pH after *H. pylori* eradication therapy recommended to infected subjects. Considering that this condition can act as stressor for this pathogen, it would be extremely informative to assess whether it affects bacterial HSP expression. Moreover, exposure of *H. pylori* cells to antibiotics, like kanamycin or chloramphenicol, leads to HspR downregulation, while HrcA expression was slightly affected only by kanamycin treatment [33]. In addition to these antibiotics, the effect of other drugs and compounds present in the *H. pylori* niche (i.e., probiotics, antioxidants) on HSP expression is still unknown and requires further studies to be elucidated.

## 3. Posttranscriptional Regulation

In addition to positive and/or negative transcriptional regulation operated by dedicated transcriptional regulatory proteins, the expression of heat-shock genes is commonly regulated also at the posttranscriptional level. In this respect, the mechanism most often employed by bacteria involves the action of chaperone proteins themselves and the functional interaction between the heat-shock σ^32^ and GroE/DnaKJE machineries in *E. coli* is so far the best-characterized example [2,34]. In this model organism the activity and the stability of the alternative sigma factor is dependent on the interaction with GroELS and DnaK proteins. During normal conditions of growth, chaperone proteins are free to interact with (and sequester) σ^32^, normally present in low amounts. Moreover, following the formation of this interaction complex, σ^32^ is delivered to protease-mediated degradation, with the protease FtsH playing the major role. When unfolded proteins accumulate in the cytoplasm, they bind to and sequester chaperone proteins which, in turn, allow σ^32^ to dissociate and accumulate as a stable protein. This feedback mechanism of homeostatic control of σ^32^ activity and stability, in which the degree of sequestration of DnaK and GroE chaperones determines the level of heat-shock genes’ transcription, links the transcriptional response to the folding state of cellular proteins. However, similar feedback regulatory strategies of chaperones modulating heat-shock transcriptional repressors have been described in different bacterial species, including *B. subtilis*, *Chlamydia trachomatis*, *S. coelicolor*, and *Mycobacterium tuberculosis* [35,36,37,38]. In these systems, chaperone proteins modulate the DNA-binding capabilities of transcriptional regulators rather than their availability or stability. The best-characterized posttranscriptional regulatory circuits involving chaperones and transcriptional regulators have been characterized for *B. subtilis* HrcA-GroE and for *S. coelicolor* HspR-DnaK functional interactions [35,37]. In these prototypical examples, during normal growth the interaction between the heat-shock repressor (HrcA or HspR) and the molecular chaperone (GroE or DnaK) stimulates the DNA-binding activity of the repressors, thereby favoring the repression of heat-shock gene transcription. As for σ^32^ regulation, chaperones sequestration by unfolded cytoplasmic proteins leads to concomitant loss of DNA-binding capabilities of the repressors, leading to induction of gene transcription [35,37]. 

**Figure 3:**
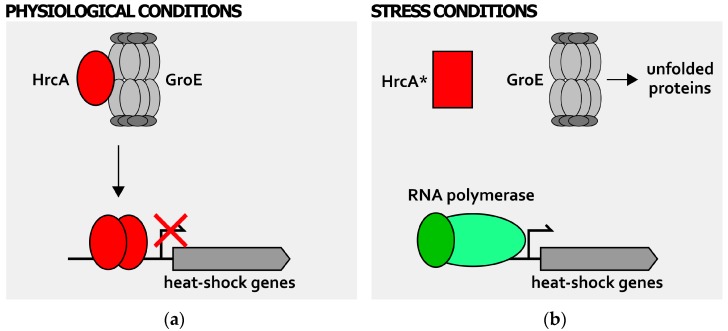
Posttranscriptional regulation of heat-shock genes expression: GroE-mediated regulation of HrcA activity. During physiological growth conditions (**a**), the GroE chaperonine (represented in grey as a multisubunit complex) is free to interact and fold the HrcA regulator (red oval). Once properly folded, this repressor binds and occupies its target promoters, repressing genes transcription; when cells experience stress conditions of growth (**b**), GroE is titrated by unfolded proteins that accumulate in the cytoplasm and cannot interact with HrcA. The repressor, left alone, has a conformation with low DNA-binding affinity (red rectangle, HrcA*) and HrcA-dependent heat-shock genes’ repression is relieved. The RNA polymerase containing the vegetative σ factor is represented by green ovals (dark green oval depicts vegetative σ factor, while light green oval represents the core RNA polymerase). Bent arrow indicates the transcription start site.

In this scenario, *H. pylori* represents a peculiar example in which two distinct chaperones modulate the activity of both HrcA and HspR transcriptional regulators. Firstly, the GroE chaperonin is able to positively affect the DNA binding affinity of HrcA for its CIRCE-like operators (Figure 3) [27,39], an observation that is in line with the GroE-HrcA functional interaction characterized in *B. subtilis* and *C. trachomatis* among the others [35,36]. According to the “titration model” proposed for *B. subtilis*, upon direct interaction with the HrcA repressor during physiological growth condition, GroE chaperonin would establish a feedback posttranscriptional regulatory circuit, enhancing HrcA DNA binding activity (Figure 3a). The accumulation of misfolded polypeptides in the cytoplasm provoked by the exposure of the cell to a stress insult would titrate away GroE, leading to a loss of HrcA DNA-binding affinity and the subsequent heat-shock promoters’ derepression (Figure 3b).

**Figure 4:**
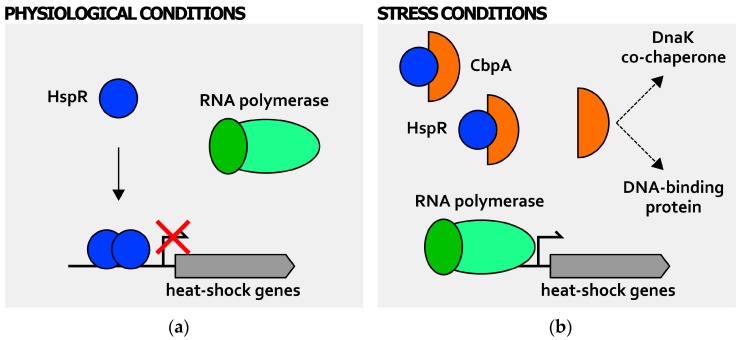
Posttranscriptional regulation of heat-shock genes expression: CbpA-dependent modulation of HspR activity. During normal growth (**a**), HspR (blue circle) binds (solid arrow) and represses heat-shock genes’ transcription by a mechanism of steric hindrance; upon stress insults (**b**), CbpA protein (orange half-circle) accumulates in the cytoplasm and interacts with HspR. This direct interaction hinders HspR DNA-binding capabilities to target promoters, whose transcription can take place. The RNA polymerase containing the vegetative σ factor is represented by green ovals (dark green oval depicts vegetative σ factor, while light green oval represents the core RNA polymerase). Bent arrow indicates the transcription start site. In analogy with *E. coli* CbpA, preliminary results suggest that CbpA of *H. pylori* possesses both co-chaperone and DNA-binding activities.

Moreover, also HspR, the second heat-shock repressor employed by *H. pylori* for heat-shock regulation, is posttranscriptionally modulated by a chaperone protein feedback. Differently from what has been observed in *S. coelicolor* and in *M. tuberculosis* [37,38], in *H. pylori* HspR DNA-binding activity is not affected by DnaK, but by the heat-shock protein CbpA (Figure 4) [40]. This protein, encoded by the first gene of the HspR-containing operon, shows a 33% amino acid sequence identity to the curved DNA binding protein A (CbpA) of *E. coli*, a DnaJ-like co-chaperone of DnaK, with also a role as a nucleoid-associated protein involved in nucleoid structuring function [41,42]. Interestingly, it has been shown that CbpA can directly interact with HspR, hindering in this way the repressor DNA-binding capabilities to target promoters (Figure 4b). 

Moreover, CbpA operates this negative regulation when the HspR repressor is in solution and with no contact with the DNA. Accordingly, cells overexpressing CbpA show deregulation of heat-shock response [40]. These findings would suggest the existence of new mechanisms of posttranscriptional regulation of heat-shock genes, as the negative effect of CbpA on HspR DNA-binding is opposite to the positive modulation operated by GroE and DnaK on HrcA and HspR, respectively. In other words, the well-established model for chaperone feedback regulation of transcriptional repressors described above is insufficient to explain CbpA-HspR functional interactions in *H. pylori*. A possibility is that CbpA regulation of HspR binding activity is required to fine-tune the shut-off response of the heat-shock genes in *H. pylori* [40]. In addition, in analogy with *E. coli* CbpA, preliminary results of our group suggest that CbpA of *H. pylori* possesses both co-chaperone and a nucleoid-associated role (Figure 4b; [43]). Consequently, it would be interesting to study in more detail the functional interplay between HspR and CbpA. That is, the heat-shock regulator HspR might influence the co-chaperone and/or nucleoid-associated activity of CbpA by direct protein-protein interaction. If confirmed, this would represent a novel example of intersection between heat-shock gene regulation and other cellular functions as, for example, the maintenance and regulation of the bacterial chromosome.

## 4. Detection of Stress Signals

The ability of bacterial cells to quickly perceive and respond to environmental perturbations that they continuously experience is crucial for survival in their living niche. A number of different stresses leads to protein unfolding and aggregation that have a severe impact on the survivability of microorganisms. Following stress signal detection, heat-shock genes are promptly transcribed and heat-shock and chaperone proteins accumulate in the cytoplasm. To accomplish the important task of connecting signal detection and genes regulation, bacteria employ a collection of specific biomolecules, whose function is to detect environmental cues and transduce them into coordinated gene expression patterns. 

In general, all classes of biomolecules can be used to measure changing environmental conditions, in particular temperature variations, including nucleic acids (DNA and RNA), proteins and lipids [2,18,44]. Nucleic acid sensing is based on the fact that these molecules can adopt different structures in response to different temperatures. For example, some mRNA molecules are characterized by specific secondary structures in the 5′-regions that are able to fold in different conformations in response to small temperature fluctuations. The general mechanism adopted by these *cis*-regulatory elements is based on the formation of a zipper-like secondary structure at permissive temperature, hindering sequence elements important for translation initiation and thereby lowering translation efficiency. Following a temperature increase, the mRNA secondary structure resolves and the rate of translation is enhanced [44,45]. This mechanism of thermoregulation assures an extremely fast response upon signal activation, acting on the pool of mRNA already present inside the cell. The DNA of the bacterial cell can also detect temperature variations. Specifically, environmental temperature can affect local DNA structures and, when these regions are proximal to promoters, the DNA conformational variation can be transduced into a modulation of transcription of neighboring genes. Transcriptional modulation takes place when the temperature-dependent local DNA structure affects RNA polymerase or transcription regulatory proteins’ interaction with promoter regions [46,47]. Bacteria can also detect temperature variations employing proteins as heat-sensors, with transcriptional regulators and chaperones being primarily involved in the context of heat-shock response. Direct heat-sensing by transcriptional regulators, a rare function for a regulator of transcription initiation and observed just in few cases [48,49,50,51], is based on the temperature-dependent DNA-binding properties of this kind of biomolecules. Specifically, the heat-sensing regulators described so far appear to be competent for promoter binding only at the permissive growth temperature. Following a temperature increase, they undergo a conformational rearrangement that lower their DNA-binding capacity [2]. 

**Figure 5:**
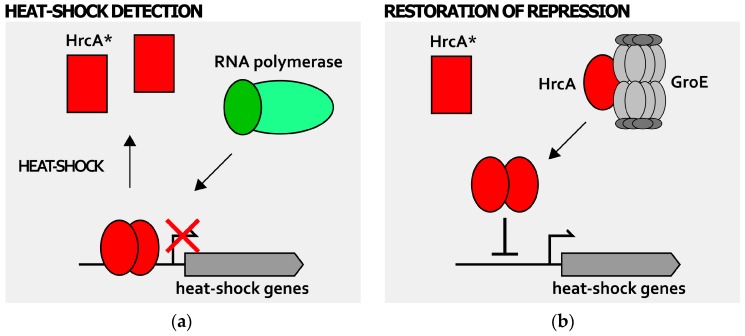
Direct temperature detection by HrcA. At 37 °C, HrcA binds its operators that overlap the core promoter of P*gro* and P*hrc* and, in combination with HspR, they repress gene transcription. Upon temperature upshift (**a**), HrcA (red oval) goes through a major structural change and acquires an inactive conformation (represented by a red rectangle and here named HrcA*), detaching from its operator (this event is depicted by a solid arrow) and relieving transcriptional repression; when the temperature returns to the physiological level (**b**), HrcA directly interacts with the GroE chaperonin and regains its native conformation (red oval), retrieving its DNA-binding capabilities (solid arrow) and restoring transcriptional repression (solid hammerhead). The RNA polymerase containing the vegetative σ factor is represented by green ovals (dark green oval depicts vegetative σ factor, while light green oval represents the core RNA polymerase). Bent arrow indicates the transcription start site.

Recent findings demonstrate that thermoregulation in *H. pylori* is directly mediated by HrcA. In particular, the heat-shock repressor HrcA has the capacity to directly sense temperature fluctuations (Figure 5). An increase of just a few degrees above 37 °C of the environmental temperature, in fact, specifically provokes a global and irreversible unfolding of HrcA, thereby inducing a complete loss of its DNA-binding activity [39]. Accordingly, upon heat-shock and loss of HrcA DNA-binding affinity for its operators, the transcription of chaperones genes is rapidly derepressed (Figure 5a). 

Intriguingly, in vitro experiments suggest that heat-exposed HrcA is irreversibly inactivated [39]. Thus, the behavior of *H. pylori* HrcA is in contrast with the properties of other intrinsic heat-sensing transcriptional regulators, whose temperature-induced loss of DNA-binding affinity is transient and reversible [48,49,50,51]. However, in vivo data suggest that HrcA would be able to recover its repressive function after the temperature challenge [39]. Further characterization of *H. pylori* heat-shock regulation revealed the role of GroE chaperonin in mediating the recovery of HrcA proper folding and DNA-binding capabilities (Figure 5b). Hence, upon heat-stress, HrcA loses DNA binding activity as a consequence of a major structural change, leading to heat-shock and chaperone genes massive transcription. When the environmental temperature lowers to a physiological level, the GroE chaperonin can interact and refold at least a fraction of heat-inactivated HrcA. Following this event, the transcriptional repression of the HrcA target promoter is restored by the refolded regulator, as well as by newly-synthetized HrcA (Figure 5b). Considering the central role of GroE chaperonin in modulating HrcA DNA-binding activity upon heat-challenge, this interaction could be exploited by *H. pylori* to adjust the transcriptional regulation in response to various stress signals. In other words, depending on the intensity of a particular stress stimulus experienced by the bacterial cell, the fraction of GroE chaperonin free to interact with HrcA will vary, influencing, in turn, the magnitude of HrcA-dependent target gene repression. In addition, the combined sensing system described above, which involves direct sensing (HrcA intrinsic temperature-sensing) and indirect sensing (GroE-mediated sensing of unfolded proteins amounts), could allow *H. pylori* to respond differentially, depending on the intensity and on the kind of stress perceived. While HrcA acts as an intrinsic protein thermometer, its partner heat-shock repressor HspR appears to be a rather stable protein, whose conformation and DNA binding activity are not influenced by temperature fluctuations [39]. Considering that the *cbpA-hspR-helicase* operon, repressed exclusively by HspR binding to P*cbp* promoter, is rapidly and strongly derepressed by heat-shock and other stresses [30], the current hypothesis is that HspR sensing mechanism maybe indirect, probably mediated by an interacting partner, whose identity is, so far, still unknown.

## 5. Genome-Wide Studies: HspR and HrcA Regulons

In order to identify additional genes regulated by HrcA and/or HspR, besides the well-characterized heat-shock and chaperone targets, genome-wide gene expression studies have been applied in the last 20 years in several bacterial species [52,53,54,55,56]. Moreover, in some instances, these whole transcriptomic studies have been combined with in vitro and in vivo DNA-binding studies, as well as with the bioinformatics search of conserved CIRCE and HAIR elements. Overall, these studies suggest that HrcA and HspR regulate, directly or indirectly, the transcription of a large number of genes involved in disparate cellular processes and not strictly related to heat-shock response. However, excluding pleiotropic effects due to *hspR* or *hrcA* disruption, these repressors seem to have a restricted direct “core regulon”, binding to and controlling the transcription of a limited set of promoters. Similarly, comparative transcriptome analysis of the wild-type strain and *hspR* and *hrcA* singly- and doubly-deficient strains revealed that in *H. pylori* these heat-shock repressors affect transcription of 43 genes in either a positive or a negative fashion. Deregulated genes encode proteins involved in different cellular processes, including stress response, iron metabolism, transport across membranes, and motility [27]. Interestingly, in the same work it has been shown that *hspR* gene inactivation led to a significant down-regulation of many genes encoding proteins involved in the regulation and biosynthesis of the flagellar apparatus. Accordingly, the *hspR*-mutant strain showed impaired motility on soft agar plates [27], suggesting the existence of an intimate interconnection between the stress response and motility functions in *H. pylori*. A similar scenario has been described in the closely related bacterium *Campylobacter jejuni* [57]. An intriguing hypothesis to explain the intersection of heat-shock genes’ regulation and motility functions could be that the aberrant accumulation of one or more components of the HspR regulon (for example GroE or DnaK chaperone proteins) could interfere with the highly-regulated assembly of some flagellar structures which, in turn, establish a proper transcriptional response. To understand more in detail the regulation of the heat shock response in *H. pylori* and to further characterize the HspR direct regulon, a recent work combined global transcriptome analysis (RNA-sequencing) with chromatin immunoprecipitation coupled to deep sequencing (ChIP-sequencing) of HspR genomic binding sites. Intriguingly, these analyses confirmed the involvement of HspR in the regulation of different crucial cellular functions. However, the number of HspR genomic binding sites identified in vivo is very restricted, being limited to the promoter region of the heat-shock operons [58], in agreement with previous observations made in several distant bacterial species [52,53,54,55,56,57].

## 6. Conclusions

Bacterial cells respond to adverse environmental growth conditions by inducing the synthesis of a class of highly conserved heat-shock proteins, which provide protection against sudden cellular damages. The major human pathogen *H. pylori* employs a sophisticated gene regulatory network, orchestrated by the two transcriptional repressors, HspR and HrcA, to finely modulate heat-shock proteins expression to defend itself from the adverse conditions during the infectious process and the long persistence in the human stomach. As detailed above, an additional level of control is exerted by chaperones themselves, through direct protein-protein interaction with the transcriptional regulators and modulation of their DNA-binding activity. *H. pylori* harbors an almost complete collection of genes coding for chaperones as well as for proteases, whose amount is expected to be enhanced upon different stress cues. Intriguingly, differently from what has been observed in many other bacterial species, protease gene promoters do not apparently belong to HrcA and/or HspR regulons, nor appear to be stress responsive [27,58]. An interesting hypothesis considers the existence of post-transcriptional or post-translational strategies, such as the presence of mechanisms of regulation acting at the RNA level (RNA thermometers), which would provide enhanced levels of these crucial players during adverse conditions of growth. 

Even though the focus of the present review is on the regulation of *H. pylori* HSP, another aspect that deserves further comments concerns the effect of *H. pylori* infection on the expression of the major gastric epithelial HSP. Surprisingly, as anticipated above, it has been shown that, upon *H. pylori* infection, the expression of some important HSP is downregulated in the host. HSP downregulation has been observed in the *H. pylori* mouse model, as well as using different gastric cancer cell lines, and different mouse and *H. pylori* strains [15]. The most prominent effect has been observed on HSP70, whose mechanism of downregulation upon *H. pylori* infection has been investigated more in detail. Specifically, in human gastric epithelium exposed to *H. pylori*, HSF-1 (heat-shock factor 1) is sequestered in a complex with phosphorylated STAT-3 protein. This interaction prevents HSF-1-mediated transcriptional activation of HSP70 under stress conditions. An additional effect of *H. pylori* infection has been observed on bax/bcl-2 cellular equilibrium, which is shifted towards a pro-apoptotic state (bax) and gives rise to apoptosis induction in the gastric epithelium [59]. These observations parallel with more recent data which demonstrate an *H. pylori*-mediated upregulation of COX-2 (Cyclooxygenase 2) mRNA expression in gastric cancer cells which, in turn, could contribute to the increase of prostaglandins generation and apoptosis enhancement [60].

In the context of host-pathogen interaction, an interesting hypothesis concerns the possible existence of reciprocal influence between bacterial and eukaryotic HSP following *H. pylori* infection of the human stomach. Some recent data, in fact, highlight that the presence of gastric epithelial cells (AGS cells) significantly increased expression levels of several transcription factors, including HspR and HrcA [33]. The molecular mechanisms responsible for the regulation of the expression of transcriptional regulators upon cell contact are still unknown and further studies on a possible functional interplay between HSP of the interacting organisms would be of great interest.

## Abbreviations

HSP	Heat-Shock Proteins
HAIR	HspR Associated Inverted Repeat
CIRCE	Controlling Inverted Repeat of Chaperone Expression
bp	Base-pairs
P*gro*	Promoter region controlling *groES-groEL* operon transcription
P*hrc*	Promoter region controlling *hrcA-grpE-dnaK* operon transcription
P*cbp*	Promoter region controlling *cbpA-hspR-helicase* operon transcription
DNase I	Deoxyribonuclease I
STAT-3	Signal transducer and activator of transcription 3
COX-2	Cyclooxygenase 2
